# Mediastinal drainage combined with upper mediastinal re-tunneling vs. mediastinal drainage alone in McKeown esophagectomy of esophageal cancer: a retrospective study

**DOI:** 10.3389/fsurg.2024.1436176

**Published:** 2024-10-02

**Authors:** Lei Dai, Xiang Tan, Mingwu Chen, Huajian Peng, Yongyong Wang

**Affiliations:** Department of Cardiothoracic Surgery, The First Affiliated Hospital of Guangxi Medical University, Nanning, China

**Keywords:** esophageal carcinoma, McKeown esophagectomy, anastomotic leakage, mediastinal drainage, upper mediastinal re-tunneling

## Abstract

**Background:**

Although mediastinal drainage may lower the risk of anastomotic leakage, the incident rate of anastomotic leakage is still high. The current study aimed to compare the effects of mediastinal drainage combined with upper mediastinal re-tunneling with mediastinal drainage only on anastomotic leakage after McKeown esophagectomy for esophageal cancer.

**Methods:**

From October 2018 to March 2021, 52 patients diagnosed as esophageal carcinoma were included in the study. 21 patients received mediastinal drainage combined with upper mediastinal re-tunneling (re-tunneling group) and 31 received mediastinal drainage only (standard group) after McKeown esophagectomy. The incidence rate of anastomotic leakage, mediastinal infection, chylothorax, thoracic infection, the peak value of leukocyte count and the mortality related to anastomotic leakage were compared between the two groups.

**Results:**

One (4.8%) patient in the re-tunneling group developed anastomotic leakage, and no patient experienced mediastinal infection or thoracic infection. Four (12.9%) patients in the standard group developed anastomotic leakage, and all these patients experienced mediastinal infection and thoracic infection (*p* < 0.05). The drainage volumes of patients in the re-tunneling group and the standard group were (170 ± 60) ml and (155 ± 45) ml, respectively, with no significant difference between the two groups (*p* > 0.05). The peak values of leukocyte count and temperature in the re-tunneling group were (14.28 ± 1.12) × 10^9^/L and (38.6 ± 1.1) °C, both lower than that of the standard group[ (16.48 ± 1.15) × 10^9^/L and (38.9 ± 1.2) °C, respectively]. But the difference was not statistically significant (*p* > 0.05). No anastomotic leakage related death occurred in both groups.

**Conclusion:**

Mediastinal drainage combined with upper mediastinal re-tunneling after McKeown esophagectomy for esophageal cancer may decrease the risk of anastomotic leakage, mediastinal and thoracic infection, reduce the inflammatory response of patients, but did not increase the mortality related to anastomotic leakage.

**Trial registration:**

The study was retrospectively registered.

## 1 Background

**Figure 1:**
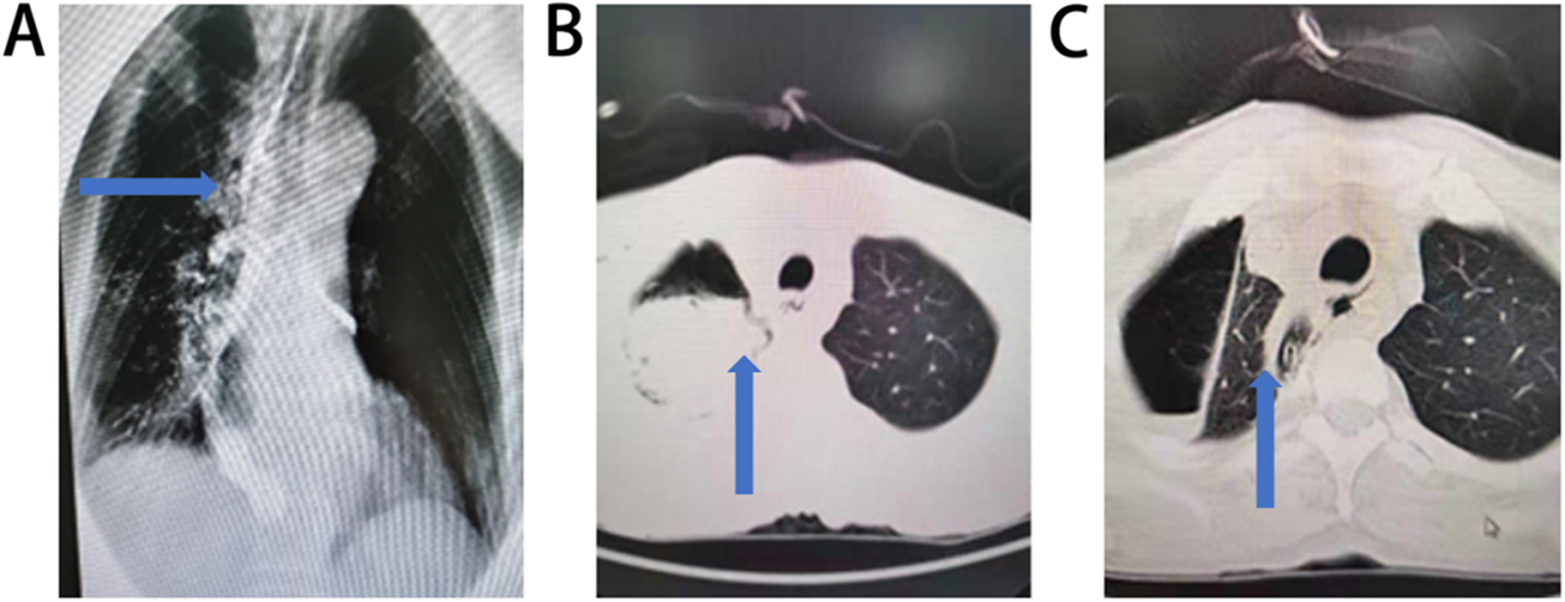
Anastomotic leakage and related complications after mcKeown esophagectomy. **(A)** Anastomotic fistula complicated with esophagotracheal fistula (The esophagotracheal fistulan was indicated by the arrow). **(B)** Anastomotic fistula complicated with thoracic infection (Thoracic infection was indicated by the arrow). **(C)** Mediastinal and thoracic infection with mediastinal drainage in a patient underwent McKeown esophagectomy and mediastinal drainage (Mediastinal and thoracic infection was indicated by the arrow).

Esophageal carcinoma is one of the most common malignant tumors in the world (1). Esophagectomy and systematic lymph node dissection remains the primary treatment for esophageal carcinoma (2). With the development of minimally invasive techniques, it has been indicated that minimally invasive esophagectomy (MIE) has the advantages of less impact on respiratory function, less trauma and rapid recovery after operation (3). McKeown esophagectomy is one of the most common used procedures for MIE (4). But the incident rate of anastomotic leakage and related complications after McKeown esophagectomy is still high (Figure 1). It was reported that the incident rates of anastomotic leakage, chylothorax and lung infection were about 14.7%, 4.9% and 11.9%, respectively (1). Though mediastinal drainage may lower the risk of anastomotic leakage, the incident rate of anastomotic leakage is still as high as 11.4% (5).

To lower the risk of anastomotic leakage and other related complications, we used a technique of mediastinal drainage combined with upper mediastinal re-tunneling after McKeown esophagectomy in esophageal carcinoma patients. In the current study, we compared the incidence rate of anastomotic leakage and related complications between this technique (re-tunneling group) and standard McKeown esophagectomy (standard group).

## 2 Methods

### 2.1 Patients

The current study is a retrospective study and aims to compare the effects of mediastinal drainage combined with upper mediastinal re-tunneling vs. mediastinal drainage only on anastomotic leakage after McKeown esophagectomy for esophageal cancer. Patients treated with treated with McKeown esophagectomy from October 2018 to March 2021 were screened., The inclusion criteria were as follow: (1) pathologically proved thoracic esophageal squamous cell carcinoma, (2) treated with McKeown esophagectomy, (3) no metastasis. If the patients had cervical esophageal carcinoma, contraindication for McKeown esophagectomy or severe organ dysfunction, they were excluded.

### 2.2 Ethics approval and consent to participate

The current study is a retrospective study and is approved by *The Medical Ethics Committee of First Affiliated Hospital of Guangxi Medical University* on June 08, 2023. The approval ID is 2023-E274-01. Written informed consent was obtained from all the participants.

### 2.3 Surgical procedure

**Figure 2:**
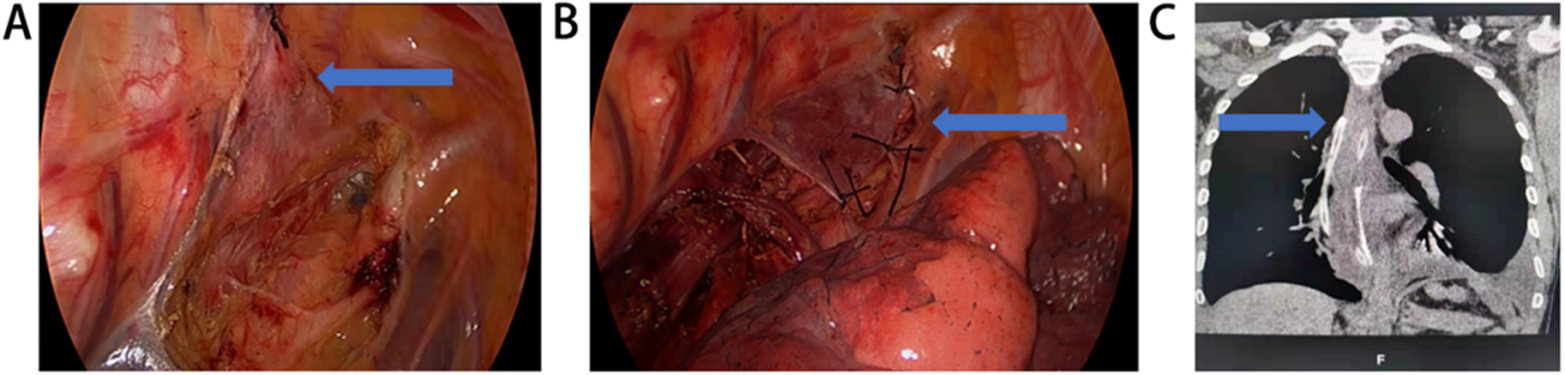
Mediastinal drainage combined with upper mediastinal re-tunneling after mcKeown esophagectomy for esophageal cancer. **(A)** The upper mediastinal pleura was unilaterally cut and sutured to the chest wall without resection (The arrow showed the upper mediastinal pleura sutured to the chest wall). **(B)** After the upper thoracic esophagus was dissected and lymph node dissection was completed, the upper mediastinal pleura was intermittently sutured to its original position and fixed, so that a tunnel was formed between the pleura and the mediastinum (The arrow showed the upper mediastinal pleura sutured and fixed to its original position). **(C)** After the abdominal operation was completed, a drainage tube was placed from the assistant operational hole. The front end of the drainage tube was placed at the level of the thoracic aortic arch (The arrow showed the front end of the drainage tube).

In the standard group, patients received standard McKeown esophagectomy. The patients underwent thoracoscopic and laparoscopic partial esophagogastric resection, thoracic and abdominal lymph node dissection, and cervical anastomosis (6). In the re-tunneling group patients, the upper mediastinal pleura was unilaterally cut and sutured to the chest wall without resection (Figure 2A). After the upper thoracic esophagus was dissociated and lymph node dissection was completed, the upper mediastinal pleura was intermittently sutured to its original position and fixed, so that a tunnel was formed between the pleura and the mediastinum, and the tubular stomach can be pulled up to the cervix through the tunnel (Figure 2B). The thoracic duct was located between the aorta and the azygos vein. In our cases, there was no tumor invasion of the thoracic duct, so we did not routinely dissect the thoracic duct. After closing the thoracic cavity, the patient was changed to supine position, and the gastric tube was made by laparoscopy. After the abdominal operation was completed, a drainage tube was placed from the assistant operational hole, which was 3–4 cm above the umbilical line of the left anterior superior iliac spine and the umbilical line, and through the hiatus of the diaphragm esophagus. The drainage tube was placed in the esophageal bed along the thoracic esophagus. The front end of the drainage tube was placed at the level of the thoracic aortic arch (Figure 2C). End to side anastomosis of gastric tube and esophagus was performed with the stapler. A nasogastric tube was positioned in call cases for intracavitary monitoring and for a better drainage of secretions. All the patients were operated by the same medical team to ensure the standardization of the operation.

### 2.4 Postoperative management

All patients fasted for 7 days after surgery, received total parenteral nutrition and anti-infection treatment. Computed tomography (CT) scan was performed on the fifth day after surgery. If no anastomotic leakage or thoracic infection was found by CT scan, patients were allowed to eat on the seventh day after surgery. The criteria for drainage tube removal were: (1) drainage volume less than 200 ml/day, (2) the color of drainage fluid was light red or light yellow, (3) no symptom or other manifestation of anastomotic leakage, thoracic infection, chylothorax and other complications. If the chest CT scan or the patients’ clinical manifestation suggest anastomotic leakage, including digestive tract content emerging from the neck incision or the drainage tube, fever, severe chest pain, an upper gastrointestinal imaging with oral contrast agents was performed to confirm the anastomotic leakage.

### 2.5 Observational clinical outcomes

Patient's basic information, including age, sex, tumor location, clinical stage, were recorded. After the surgery, information of temperature, leukocyte count, drainage volume, anastomotic leakage, chylothorax, mediastinal infection and thoracic infection were recorded.

### 2.6 Statistical analysis

If parameters were continuous variables and met normality, they were reported as medians (range) or mean ± standard errors (SD). If parameters were categorical variables, they were presented as frequencies (%). The incidence rates of anastomotic leakage, mediastinal infection, chylothorax, thoracic infection, the peak value of leukocyte count and the mortality related to anastomotic leakage were compared between the two groups. Data were analyzed and compared using Chi square test and *t*-test. The analysis was performed by SPSS 19.0 software. If *p* < 0.05, the difference was considered as significant.

## 3 Results

### 3.1 Patient characteristics

From October 2018 to March 2021, 52 patients diagnosed as esophageal carcinoma were included in the study. 21 patients treated with mediastinal drainage combined with upper mediastinal re-tunneling after McKeown esophagectomy (re-tunneling group). 31 patients treated with mediastinal drainage only after McKeown esophagectomy (standard group). Patient characteristics of the two groups were shown in Table 1. There was no statistical difference between the two groups in age, gender, tumor location, clinical stage, neoadjuvant chemotherapy history and Histopathology type (*p* > 0.05). No patient received neoadjuvant radiotherapy in both group.

**Table 1:** Patient characteristics of the two groups.

Characteristics	Re-tunneling group (*N* = 21)	Standard group (*N* = 31)	*P*
Age (*n*, %)			0.77
Median (IQR)	53 (47–73)	54 (45–69)	
<60 years	12 (57.1)	20 (64.5)	
>60 years	9 (42.9)	11 (35.5)	
Gender (*n*, %)			>0.99
Male	17 (80.9)	24 (77.4)	
Female	4 (19.1)	7 (22.6)	
Tumor location (*n*, %)			0.51
Upper thoracic	1 (4.7)	0 (0)	
Middle thoracic	15 (71.4)	21 (67.7)	
Lower thoracic	5 (23.8)	10 (32.3)	
Stage (*n*, %)			0.50
IA–IB	4 (19.0)	3 (9.7)	
IIA–IIB	15 (71.4)	22 (70.9)	
IIIA	2 (9.5)	6 (19.4)	
Neoadjuvant chemotherapy (*n*, %)			
Yes	12 (57.1)	17 (54.8)	>0.99
No	9 (42.9)	14 (45.2)	
Histopathology type			
Adenocarcinoma	0 (0)	0 (0)	–
Squamous cell carcinoma	21 (100)	31 (100)	

IQR, Interquartile range.

### 3.2 Incidence rates of anastomotic leakage, mediastinal infection and thoracic infection was lower in the re-tunneling group

One (4.8%) patient in the re-tunneling group and four (12.9%) patients in the standard group had anastomotic leakage (*p* < 0.05). The statistical power was 0.82. No patient in the re-tunneling group had mediastinal infection or thoracic infection, but four (12.9%) patients in the standard group developed mediastinal infection and thoracic infection (*p* < 0.05). No chylothorax occurred in both groups of patients (Table 2).

**Table 2:** Postoperative complications and other parameters of the two groups.

	Re-tunneling group (*N* = 21)	Standard group (*N* = 31)	*P*
Drainage volume(mean ± SD, ml)	170 ± 60 ml	155 ± 45 ml	0.83
Anastomotic leakage (*n*, %)	1, 5.8%	4, 12.9%	<0.03
Chylothorax (*n*, %)	0, 0.0%	0, 0.0%	–
Mediastinal infection (*n*, %)	0, 0.0%	4, 12.9%	0.02
Thoracic infection (*n*, %)	0, 0.0%	4, 12.9%	0.02
Peak value of leukocyte count^a^ (mean ± SD, ×10^9^/L)	14.28 ± 1.12	16.48 ± 1.15	0.21
Peak value of temperature^b^ (mean ± SD, °C)	38.6 ± 1.1	38.9 ± 1.2	0.11
Death (*n*, %)	0, 0.0%	0, 0.0%	–

^a^ Maximum leukocyte count within 1 week after surgery.

^b^ Highest body temperature within 1 week after surgery.

### 3.3 The drainage volumes, peak values of leukocyte count, and temperature did not differ significantly between the two groups

The drainage volumes of patients in the re-tunneling group and the standard group were (170 ± 60) ml and (155 ± 45) ml, respectively, with no significant difference between two groups (*p* > 0.05). The peak values of leukocyte count and temperature in the re-tunneling group were (14.28 ± 1.12) × 10^9^/L and (38.6 ± 1.1) °C, both lower than that in the standard group [ (16.48 ± 1.15) × 10^9^/L and (38.9 ± 1.2) °C, respectively]. But the difference was not statistically significant (*p* > 0.05). No chylothorax or anastomotic leakage related death occurred in both groups (Table 2).

## 4 Discussion

Surgery combined with radiotherapy and chemotherapy is the primary treatment for esophageal cancer (7). McKeown esophagectomy is one of the most widely used surgical procedures (1). Anastomotic leakage is a common and severe complication of McKeown esophagectomy that can be fatal. It was reported that the incident rate of anastomotic leakage after McKeown esophagectomy was about 9.2%–23.9% (8, 9). High body mass index (BMI), cervical anastomosis, diabetes mellitus, COPD, decreased postoperative albumin and postoperative renal dysfunction are potential risk factors for anastomotic leakage (9, 10). Anastomotic leakage prolongs hospital stays, increases treatment costs and it is an independent risk factor for tumor recurrence (11, 12). The digestive juice can enter the chest cavity through the leakage, causing acute pleurisy reaction and serious chest infection, resulting in systemic infection and other related symptoms such as electrolyte disturbance and high fever. In serious cases, multiple organ failure may occur and even lead to death (13). The placement of mediastinal drainage tube is an effective way for early detection of anastomotic leakage and facilitates the leakage healing, and thus help to reduce the risk of death and improve the prognosis of patients (14). However, the mediastinal drainage tube does not reduce the risk of anastomotic leakage (14, 15).

**Figure 3:**
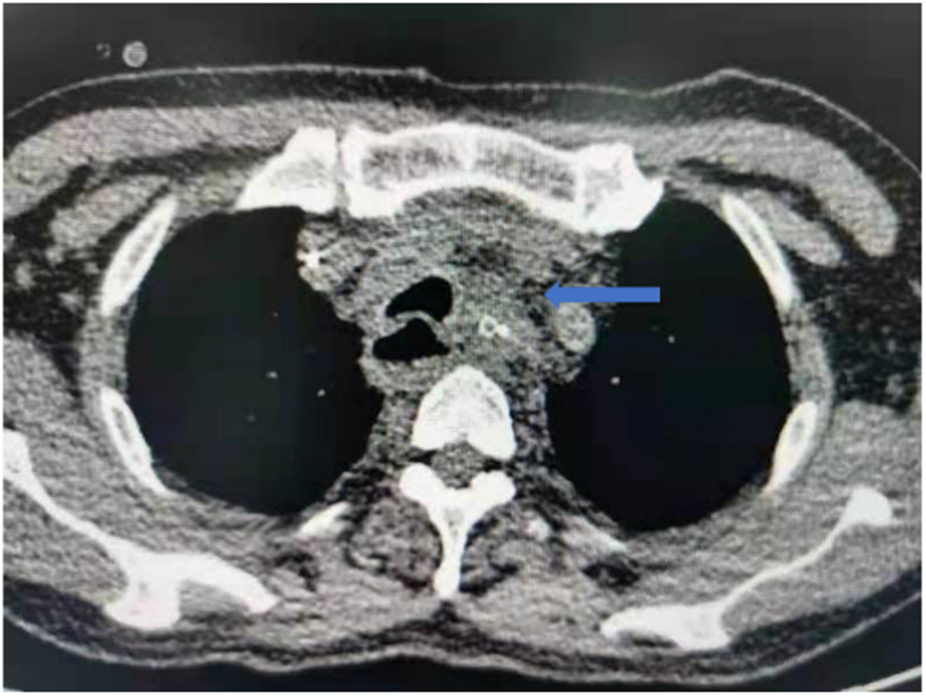
One patient in the re-tunneling group had anastomotic leakage, but the patient did not experience with mediastinal and thoracic infection.

In the current study, we used a mediastinal drainage combined with upper mediastinal re-tunneling technique after McKeown esophagectomy and showed that this technique may help to reduce the incident rate of anastomotic leakage. In the re-tunneling group, one patient had anastomotic leakage and the patient did not complicate with mediastinal or thoracic infection (Figure 3). In this case, the leaked digestive juice was discharged from the neck incision. Only a small amount of the leaked digestive juice entered the upper mediastinum, for that the upper mediastinal pleura was sealed. The digestive juice in the upper mediastinum can be led out by the mediastinal drainage tube and therefore did not cause mediastinal infection or thoracic infection. The incident rate of anastomotic leakage in the re-tunneling group was significantly lower than that in the standard group. We considered that the suture of the upper mediastinal pleura had a supporting effect on the posterior wall of the anastomosis, which reduced the tension of the posterior wall of the anastomosis, and thus reduced the incidence of anastomotic leakage. The incidence of anastomotic leakage in the standard group was 12.9%, which was consistent with previous reports (8). Due to that the sample size was determined by the number of esophageal cancer patients admitted to our center during the study period, but was not based on statistical calculations, we calculated the statistical power of the current study with the current sample size. The statistical was 0.82, which means that there is a 82% chance that the current sample size will detect the difference in incidence of anastomotic leakage between the two groups. There was no significant difference between the two groups in drainage volume, peak leukocyte count and peak temperature. One reason may be that the mediastinal drainage tube drained the inflammatory cytokines out of the body and reduced the inflammatory reaction in both groups. Another reason may be that the sample size of the current study was too small to draw a statistical difference. We also noticed that no chylothorax occurred in both groups of patients. The reason may be that in all our cases, thoracic duct was intact because we did not dissect the thoracic duct for no tumor invasion.

It was the first study about the efficacy and safety of sealing the upper mediastinal pleura at the base of McKeown esophagectomy. Some limitations of this study should be noticed. Firstly, we do not routinely perform upper gastrointestinal imaging with oral contrast agents within one week after surgery. We routinely perform chest CT scans to assess for anastomotic leakage, mainly based on chest CT images and patient manifestations. Upper gastrointestinal imaging with oral contrast agents was performed only if anastomotic leakage was suspected. This may underestimate the incidence of anastomotic leakage, especially in some mild cases. Secondly, we did not analyze other risk factors related to anastomotic leakage between the two groups, which may cause some bias. Thirdly, the sample size was small. There were only 21 patients included in the re-tunneling group and 31 patients in the standard group. The sample size was determined by the number of esophageal cancer patients admitted to our center during the study period, but not based on statistical calculations. Therefore, the statistical power may be insufficient. Fourthly, In China, only about 30% of patients with stage II or higher esophageal cancer receive neoadjuvant therapy, and most centers have not yet adopted neoadjuvant therapy as a routine practice. The center where the authors are based only began gradually implementing standardized neoadjuvant therapy starting in October 2023. Thus, the proportion of patients who received neoadjuvant therapy seems lower than expected when compared to the stage data in our study. Fourthly, all the patients were from the same medical institution and selection bias may exist. What's more, this is a retrospective study and there may be some recall bias.

## 5 Conclusion

In summary, mediastinal drainage combined with upper mediastinal re-tunneling after McKeown esophagectomy for esophageal cancer may decrease the risk of anastomotic leakage, mediastinal and thoracic infection and did not increase the mortality related to anastomotic leakage. But the benefit of this surgical method needs to be further confirmed by large sample, multi-center and prospective studies.

## Data Availability

The raw data supporting the conclusions of this article will be made available by the authors, without undue reservation.
